# Transcriptional regulation by Poly(ADP-ribose) polymerase-1 during T cell activation

**DOI:** 10.1186/1471-2164-9-171

**Published:** 2008-04-16

**Authors:** Luis Saenz, Juan J Lozano, Rut Valdor, Alberto Baroja-Mazo, Pablo Ramirez, Pascual Parrilla, Pedro Aparicio, Lauro Sumoy, José Yélamos

**Affiliations:** 1 Transplant Unit, Department of Surgery, University Hospital "Virgen de la Arrixaca", University of Murcia, Ciberehd, Murcia, Spain; 2 Bioinformatics and Genomics Program, Centre for Genomic Regulation, PRBB, Barcelona, Spain; 3 Department of Biochemistry, Molecular Biology and Immunology, University of Murcia, Murcia, Spain; 4 Department of Immunology, IMIM-Hospital del Mar, PRBB, Barcelona, Spain; 5 Plataforma de Bioinformática, Centro de Investigación Biomédica en red de Enfermedades Hepáticas y Digestivas (CIBERehd), Hospital Clinic, Barcelona, Spain

## Abstract

**Background:**

Accumulating evidence suggests an important role for the enzyme poly(ADP-ribose) polymerase-1 (PARP-1) as an integral part of the gene expression regulatory machinery during development and in response to specific cellular signals. PARP-1 might modulate gene expression through its catalytic activity leading to poly(ADP-ribosyl)ation of nuclear proteins or by its physical association with relevant proteins. Recently, we have shown that PARP-1 is activated during T cell activation. However, the proposed role of PARP-1 in reprogramming T cell gene expression upon activation remains largely unexplored.

**Results:**

In the present study we use oligonucleotide microarray analysis to gain more insight into the role played by PARP-1 during the gene expression reprogramming that takes place in T cells upon activation with anti-CD3 stimulation alone, or in combination with anti-CD28 co-stimulation. We have identified several groups of genes with expression modulated by PARP-1. The expression of 129 early-response genes to anti-CD3 seems to be regulated by PARP-1 either in a positive (45 genes) or in a negative manner (84 genes). Likewise, in the presence of co-stimulation (anti-CD3 + anti-CD28 stimulation), the expression of 203 genes is also regulated by PARP-1 either up (173 genes) or down (30 genes). Interestingly, PARP-1 deficiency significantly alters expression of genes associated with the immune response such as chemokines and genes involved in the Th1/Th2 balance.

**Conclusion:**

This study provides new insights into changes in gene expression mediated by PARP-1 upon T cell activation. Pathway analysis of PARP-1 as a nuclear signalling molecule in T cells would be of relevance for the future development of new therapeutic approaches targeting PARP-1 in the acquired immune response.

## Background

T lymphocyte activation requires recognition through the antigen-specific T cell receptor (TCR)/CD3 complex of foreign peptides presented by self-MHC molecules on antigen presenting cells in context with co-stimulatory signals mediated by co-stimulatory receptors such as CD28 [1]. The concurrent ligation of these receptors initiates signal transduction pathways that lead to the induction of a complex array of kinases and phosphatases, and the downstream activation of transcription factors such as NF-κB, AP-1 and NFAT which play a critical role in reprogramming gene expression [2]. The overall result is the proliferation, differentiation and activation of T cells. However, T cells stimulated via the TCR/CD3 complex alone do not become fully activated and can become anergic or even apoptotic [3]. Transcriptional changes during T cell stimulation are tightly regulated by a variety of mechanisms involving not only interactions between a complex network of transcription factors and cis-regulatory DNA regions, but also epigenetic changes in chromatin structure including acetylation, phosphorylation, methylation and ubiquitylation [4].

Accumulating evidence suggests an important role for the enzyme poly(ADP-ribose) polymerase-1 (PARP-1) in the regulation of gene expression during development and in response to specific cellular signals [5,6] working at different levels. PARP-1 belongs to a family of enzymes (PARP) that, using NAD^+ ^as a substrate, synthesize and transfer homopolymers of ADP-ribose onto glutamic acid residues of acceptor proteins mainly involved in chromatin structure and DNA metabolism. Poly(ADP-ribosyl)ation is terminated by the release of extensively poly(ADP-ribosyl)ated (negatively charged) PARP molecules from DNA. ADP-ribose polymers are then subjected to degradation by poly(ADP-ribose) glycohydrolase (PARG) [6]. Poly(ADP-ribosyl)ation is therefore an immediate, covalent, but transient post-translational modification of cellular proteins playing and important role in epigenetic regulation of chromatin structure and gene expression under physiological conditions in which the integrity of the DNA is maintained [6]. PARP-1 activity, responsible for almost 90% of poly(ADP-ribosy)lation in the cell, might modulate gene expression through poly(ADP-ribosyl)ation of its partner proteins or by its physical association with relevant proteins such as transcription factors. Regulation by PARP-1 has been described for different transcription factors such as NF-κB [7,8], E2F-1 [9], C/EBPalpha [10], YY-1 [11], RNA polymerase II-associated factors [12], p53 [13] and NFAT [14]. Indeed, PARP-1 and poly(ADP-rybosyl)ation play a critical role in the expression control of multiple NF-κB dependent genes involved in the inflammatory response [15]. In addition, different studies have analysed the effects of PARP-1 deficiency on gene expression in fibroblasts [16-18], cardiomyocytes [19], leukemia cells [20], glia [21], endothelial cells [15], embryonic stem cell lines and liver tissue [22] at the genome-wide level.

Recently, we have demonstrated that PARP-1 is activated during T cell activation, modulating the activity of the NFAT transcription factor [14]. In the present study we use oligonucleotide microarray analysis to gain more insight into the role played by PARP-1 during the gene expression reprogramming that takes place in T cells upon activation with anti-CD3-stimulation alone, or in combination with anti-CD28 co-stimulation. These two stimulation conditions would allow us to analyse the role of PARP-1 in the modulation of genes upon NFAT activation under conditions in which both AP-1 and NF-κB are fully activated (anti-CD3 + anti-CD28 co-stimulation) or poorly activated (anti-CD3 alone). T cells co-stimulated with anti-CD3 + anti-CD28 develop an overlapping transcription network with anti-CD3 stimulated T lymphocytes, but co-stimulatory signalling quantitatively alters components of the TCR/CD3 complex-induced signals [23]. Therefore, PARP-1 could synergize with different transcription factors in order to stimulate or repress gene expression in both stimulation conditions. We have identified several groups of genes, whose expression is modulated by PARP-1 either in a positive or a negative manner. Interestingly, PARP-1 deficiency significantly alters expression of genes associated with the immune response such as chemokines and genes involved in the Th1/Th2 balance.

## Results and Discussion

### Transcriptional programs in Parp-1^-/- ^vs Parp-1^+/+ ^T cells in response to antigen receptor stimulation

In order to study the role of PARP-1 in the transcriptional regulation of T cells, we have analysed the gene expression profiles in splenic T cells derived from PARP-1-wild-type (Parp-1^+/+^) and PARP-1-deficient (Parp-1^-/-^) mice under both resting and antigen receptor stimulation conditions, using oligonucleotide microarray technology. Total RNA prepared from resting or stimulated cells was purified, reverse transcribed and amplified by in vitro transcription in the presence of biotinylated nucleotides, and the labeled cRNA was hybridized on Affymetrix oligonucleotide chips (murine genome 430A 2.0) containing over 22600 probe sets representing transcripts from over 14000 well-characterized mouse genes. Only those differences in RNA abundance that were reproducible in independent experiments with different batches of cells and represented by a change of 1.5-fold or greater (p-value < 0.05) were considered.

At a basal level, we found 93 genes (0.66%) that differed in their expression between Parp-1^+/+ ^and Parp-1^-/- ^T cells. This percentage is quite similar to the change in the basal expression level observed between primary Parp-1^+/+ ^and Parp-1^-/- ^fibroblasts [18]. The expression of 50 of these genes in T cells was higher in Parp-1^-/- ^than in Parp-1^+/+ ^T cells (negatively regulated by PARP-1), while the expression of 43 genes, obviously including the Parp-1 gene itself, was lower in Parp-1^-/- ^compared to Parp-1^+/+ ^T cells (positively regulated by PARP-1) (Table 1). The specific genes that were differentially expressed in Parp-1^-/- ^vs Parp-1^+/+ ^T cells are listed in the Additional files 1 and 2. In addition, all data are deposited in the ArrayExpress database [24] with accession number E-MEXP-1237. We did not observe genes overlapping between the report on primary fibroblast and our present results. Similar absence of overlap has been reported recently by Ogino et al, between fibroblasts and ES cells [22]. This may be possibly due to differences in the cell linages examined in these different experiments.

**Table 1:** PARP-1 dependent genes in T cells^a^

Stimulation		No. of genes	
	Total	Positive regulation^b^	Negative regulation^b^

None	93	43	50
Anti-CD3	129	45	84
Anti-CD3+anti-CD28	203	173	30

^a ^Genes were classified as PARP-1 dependent genes when their expression was change by a factor of at least 1.5 (F-test<0.05) between Parp-1^+/+ ^and Parp-1^-/- ^cells in the microarrays analysis. ^b^Positive regulation was considered when the expression of a particular gene was higher in the presence of PARP-1 than in its absence. Likewise, genes expressed at higher level in the absence of PARP-1 than in its presence were considered negatively regulated by PARP-1.

To characterize the gene expression program in T cells responding to antigen receptor stimulation, T cells from both genotypes were subjected to stimulation for 3.5 h with a plate-bound antibody to CD3 alone, or in combination with an antibody to CD28 and expression results were normalized to the unstimulated conditions. Short stimulation times have been shown to induce important changes in T cells gene expression programs [25,26]. CD3 stimulation modulated a significant number of early-response genes (1168) (8.3% of all genes represented in the microarray) in Parp-1^+/+ ^T cells, up-regulating 755 and concomitantly down-regulating 413 transcripts, while in Parp-1^-/- ^T cells 958 of genes (6.8%) were modulated, up-regulating 656 genes and down-regulating 302 transcripts. On the other hand, CD3 stimulation in the context of CD28 co-stimulatory signal, modulated a significant number of early-response genes (1085) (7.8%) in Parp-1^+/+ ^T cells, up-regulating 722 and concomitantly down-regulating 363 transcripts, while in Parp-1^-/- ^T cells 1137 of genes (8.1%) were modulated, up-regulating 669 genes and down-regulating 468 transcripts.

### Identification of PARP-1-dependent genes during early T cell activation

Transcriptional changes during T lymphocyte activation have also been characterized in the past [25], and it is beyond the scope of this paper to provide a discussion of all of the observed changes and their biological implications. However, comparison of the gene expression programs evoked by activation in Parp-1^+/+ ^and Parp-1^-/-^T cells shows that 129 early-response genes to anti-CD3 seem to be PARP-1-dependent (fold-change >1.5; F-test p-value<0.05) and regulated by PARP-1 either in a positive (higher expression in Parp-1^+/+ ^than in Parp-1^-/- ^T cells) (45 genes) or in a negative manner (higher expression in Parp-1^-/- ^than in Parp-1^+/+ ^T cells) (84 genes) (Table 1). Likewise, in the presence of co-stimulation (anti-CD3 + anti-CD28 mAbs stimulation), the expression of 203 genes is also regulated by PARP-1 either in a positive (173 genes) or in a negative manner (30 genes) (Table 1). In addition, these data are listed in the Additional files 3 and 4. Interestingly, response to anti-CD3 alone resulted in a higher number of genes negatively regulated by PARP-1 than those regulated positively. However, anti-CD3 stimulation in the presence of anti-CD28 co-stimulation produces the opposite effect, favouring expression of genes regulated positively by PARP-1 (Table 1). These results could be related with a different role of PARP-1 in the regulation of specific transcription factors involved in those signalling pathways. Thus, T cell stimulation via the TCR/CD3 complex alone seem to be sufficient for NFAT but not for optimal NF-κB or AP-1 activation. We have recently demonstrated that PARP-1 inhibition seems to modulate in an opposite manner NFAT and NF-κB-dependent transcriptional activity in T cells, improving NFAT [14] and inhibiting NF-κB activities (data not shown). However, the complexity of the NFAT and NF-κB families, together with the regulation of genes by both transcriptional factor members precludes ascribing the described PARP-1-dependent genes to a unique and direct effect on NFAT or NF-κB dependent transcriptional activity.

**Figure 1:**
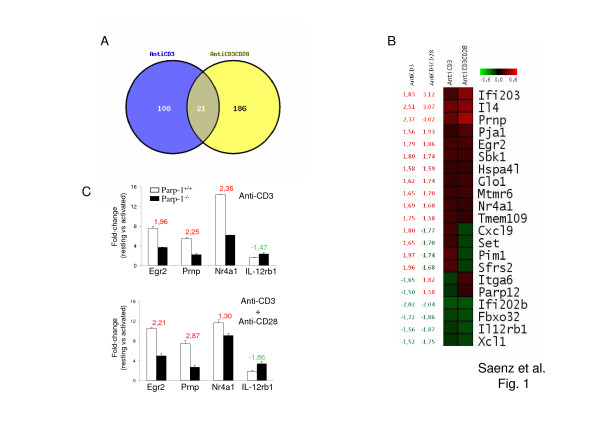
**Gene expression profile analysis of Parp-1^+/+ ^and Parp-1^-/- ^activated T cells**. (A) Venn diagram showing the numbers of PARP-1-dependent genes in T cells upon stimulation with anti-CD3 mAb alone or in combination with anti-CD28 mAb. (B) Expression maps including the genes dependent on PARP-1 in response to both anti-CD3 and anti-CD3 + anti-CD28 stimulation. Red indicates higher expression in Parp-1^+/+ ^T cells compared to Parp-1^-/- ^T cells (positive regulation by PARP-1) while green indicates higher expression in Parp-1^-/- ^T cells compared to Parp-1^+/+ ^T cells (negative regulation by PARP-1). Numbers on the left column indicate the fold-change expression in Parp-1^+/+ ^T cells compared to Parp-1^-/- ^T cells. (C) Real-time PCR analysis of representative genes. Samples were normalized according to Gapdh expression levels. The y-axis represents fold-change of activated versus resting cells for both Parp-1^+/+ ^(white bars) and Parp-1^-/- ^(black bars) T cells. Results represent the mean value ± SD of two independent experiments. The ratio of gene expression in Parp-1^+/+ ^over Parp-1^-/- ^cells (positive number) or the ratio of gene expression in Parp-1^-/- ^over Parp-1^+/+ ^cells (negative number) is indicated above each pair of bars.

Twenty one genes were dependent on PARP-1 for response to both anti-CD3 and anti-CD3 + anti-CD28 stimulation (Figures 1A and 1B). Eleven of these genes were positively regulated by PARP-1 while 4 of them were negatively regulated by PARP-1 in response to either anti-CD3 or anti-CD3 + anti-CD28 stimulation. However, 4 genes (Cxcl9, Set, Pim1, and Sfrs2) were regulated positively by PARP-1 in response to CD3 stimulation alone, but the same genes were regulated negatively by PARP-1 in the presence of co-stimulation through CD28. Likewise, 2 genes (Itga6 and Parp-12) were regulated negatively by PARP-1 in response to CD3 stimulation but positively by PARP-1 in response to anti-CD3 + anti-CD28 stimulation (Fig. 1B). We confirmed expression changes for some genes by Real-time PCR. Although there were differences in the absolute fold-change values detected by the two methods, PCR results correlated well with the differential gene expression data produced using Affymetrix GeneChips (Figure 1C).

**Figure 2:**
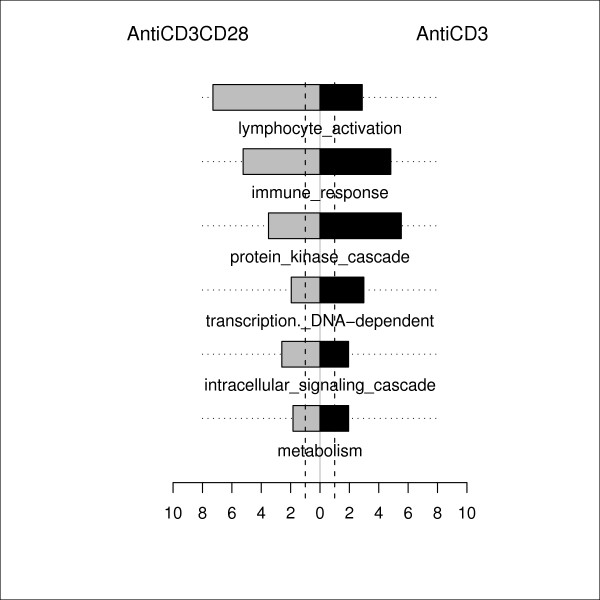
**Gene Ontology term enrichment analysis of PARP-1 dependent genes upon T cell activation**. More genes than expected by chance were involved in certain processes or functions in response to anti-CD3 stimulation alone (black bar) or in response to anti-CD3 + anti-CD28 stimulation (grey bar). The length of the bar indicates the ratio between numbers of observed PARP-1 dependent genes in each processes or functions and the number expected by chance.

Automatic functional annotation of PARP-1-dependent genes in response to either anti-CD3 or anti-CD3 + anti-CD28 stimulation was performed based on Ingenuity software. Although this type of annotation is very useful to obtain a description of the transcriptome, it can be misleading if the absolute number of genes that fall into each class is used as the only factor for selection of relevant classes. Consequently, we identified which Gene-Ontology (GO) terms were significantly over-represented in each activation condition (anti-CD3 alone or anti-CD3 in combination with anti-CD28). As shown in the examples in Figure 2, more PARP-1-dependent genes than expected by chance were involved in certain processes or functions. The bars indicate whether there are more PARP-1-dependent genes with a given GO term than expected with a random distribution in each activation condition. Interestingly, the highest differences were found for genes involved in lymphocyte activation and immune response, with little differences in genes involved in metabolism.

### PARP-1 deficiency affects the expression of genes involved in the immune response pathway in T cells

**Figure 3:**
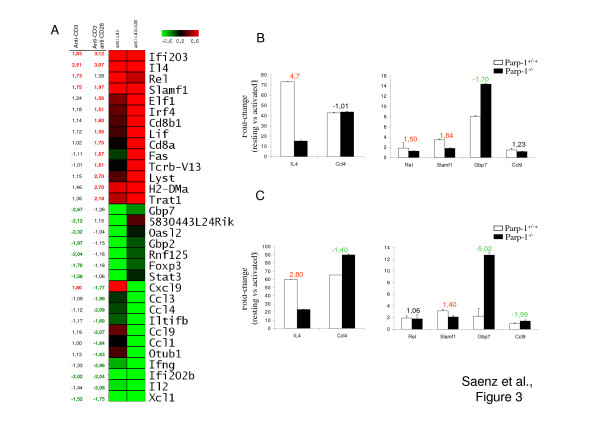
**PARP-1 dependent genes in T cells involved in the immune response**. (A) Expression maps including all the genes belonging to GO terms "immune response". Red indicates higher expression in Parp-1^+/+ ^T cells compared to Parp-1^-/- ^T cells (positive regulation by PARP-1) while green indicates higher expression in Parp-1^-/- ^T cells compared to Parp-1^+/+ ^T cells (negative regulation by PARP-1). Numbers in the left column indicate the fold-change expression in Parp-1^+/+ ^T cells compared to Parp-1^-/- ^T cells. (B) Real-time PCR analysis of representative genes in response to anti-CD3 stimulation or (C) in response to anti-CD3 + anti-CD28 stimulation. Samples were normalized according to Gapdh expression levels. The y-axis represents fold-change of activated versus resting cells for both Parp-1^+/+ ^(white bars) and Parp-1^-/- ^(black bars) T cells. Results represent the mean value ± SD of two independent experiments. The ratio of gene expression in Parp-1^+/+ ^over Parp-1^-/- ^cells (positive number) or the ratio of gene expression in Parp-1^-/- ^over Parp-1^+/+ ^(negative number) cells is indicated above each pair of bars.

A subset of PARP-1 dependent genes in response to anti-CD3 stimulation (14 out of 129; ~11%) and in response to anti-CD3 + anti-CD28 stimulation (24 out of 203; ~12%) encode for molecules involved directly in the immune response pathway (Fig. 3A) as determined using the Ingenuity software. To validate the array data, we also evaluated expression of several of these genes by quantitative Real-time PCR. Although there were differences in the absolute fold-change values detected by the two methods, PCR results correlated well with the differential gene expression data produced using Affymetrix GeneChips (Fig. 3B and 3C). Discordant results were found between the arrays and PCR for only Gbp7 gene in response to anti-CD3 + anti-CD28 stimulation.

**Figure 4:**
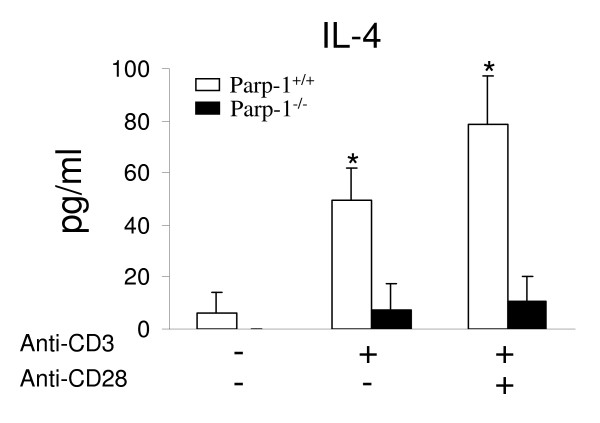
**IL-4 production in both Parp-1^+/+ ^and Parp-1^-/- ^T cells**. T cells were stimulated for 24 h with anti-CD3 or anti-CD3 + anti-CD28 mAbs. The level of IL-4 was measured by ELISA. An experiment representative of three experiments is shown. Results represent the mean value ± SD. * P < 0.05 in t-test.

In response to stimulation through CD3 alone or in combination with mAb to CD28 a significantly increased expression of IL-4 was detected in Parp-1^+/+ ^compared to Parp-1^-/- ^T cells (Fig. 3A). This result was further confirmed by Real-time PCR (Fig. 3B and 3C). In addition, T cells purified from Parp-1^+/+ ^and Parp-1^-/- ^mice were stimulated for 24 hours with anti-CD3, alone or in combination with anti-CD28 mAb, and IL-4 released in the supernatants was measured by ELISA (Fig. 4). The results verified the effect of PARP-1 deficiency on the expression levels of IL-4 obtained at the mRNA level in the microarray and Real-time PCR experiments. Overall, our data suggest an important role for PARP-1 in the regulation of IL-4 expression. IL-4 is a major Th2 effector cytokine and a key promoter of Th2 development [4]. Th1 and Th2 cells are characterized by the types of cytokines that they produced. IFN-gamma is the hallmark of Th1 cells which are involved in the immune response against intracellular microoganisms. On the other hand, Th2 effector cells secrete IL-4, IL-5 and IL-13 being involved in immune response to helminth infections as well as to IgE-dependent hypersensitivity reactions. Interestingly, the expression of IFN-gamma mRNA seem to be increased in Parp-1^-/- ^T cells. Expression of several Th1 chemokines such as Xcl1, Ccl4 and Ccl9 seem to be also increased en Parp-1^-/- ^T cells in response to anti-CD3 + anti-CD28 activation (Fig. 3). Therefore, the absence of PARP-1 seems to bias T cell response to a Th1 phenotype. However, our microarray data did not reveal any difference in the expression of other Th2 cytokines such as IL-5, IL-10 and IL-13 between Parp-1^-/- ^and Parp-1^+/+ ^T cells, suggesting that PARP-1 could specifically control IL-4 gene expression which might act on Th1 differentiation by supressing IL-12bR expression and IFN-gamma secretion. Previous studies have demonstrated the requirement of Slam in IL-4 production by T cells [27]. Similarly, Irf4 gene expression was also found to be higher in Parp-1^+/+ ^than in Parp-1^-/- ^T cells after stimulation with anti-CD3 + anti-CD28 (Fig. 3A). Irf4 seems to enhance IL-4, but not IL-2 promoter activity [28]. Whether PARP-1 controls IL-4 expression through an interaction with cis-regulatory elements or chromatin remodelling at IL-4 locus remains to be elucidated.

Reduction of IL-4 production by lung cells has been reported in OVA-challenged Parp-1^-/- ^mice compared with wild-type mice as well as in wild-type mice treated with the PARP activity inhibitors TIQ-A [29], although pharmacological inhibition of PARP activity using the PJ34 inhibitor in peripheral blood lymphocytes did not suppress IL-4 production [30]. These discrepancies might be associated to side effects of the different inhibitors used in these studies. Further studies are therefore needed to determine whether PARP-1 is a new player in the epigenetic regulation of IL-4 by T cells [4] or is involved on the immune response associated with a Th2 bias. Autoinmunity seem to be associated to an immune response bised to the differentiation and activation of Th1 and/or Th17 effectors T cells. PARP-1 seems to positively control Ifi203 expression even in the absence or presence of CD28-stimulation (Fig. 3 and data not shown). In sharp contrast, Ifi202b was preferentially expressed in Parp-1^-/- ^stimulated T cells. A high expression of Ifi202b and a low expression of Ifi203 and was also observed in B6.Nba2 autoimmune prone mice [31]. Therefore, PARP-1 could play a role in the consequence of autorecognition acting through Ifi203 and Ifi202b gene regulation.

### T cell proliferation is impaired in the absence of PARP-1

**Figure 5:**
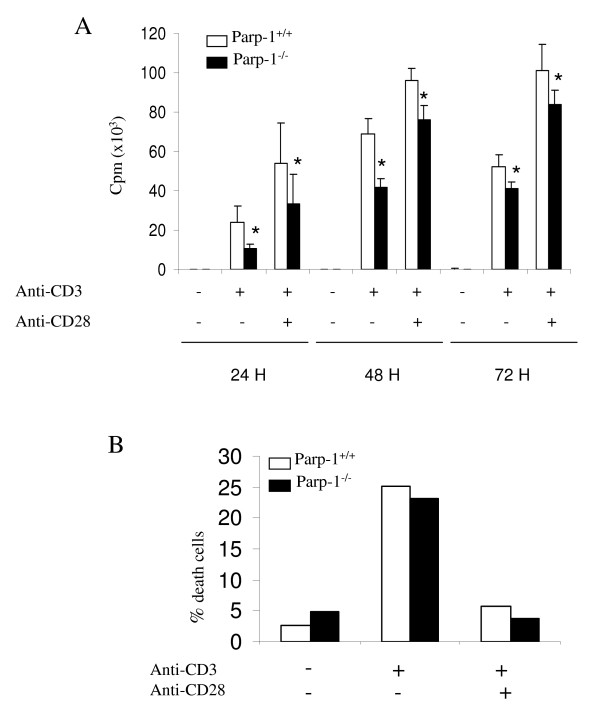
**T cell proliferation and apoptosis in Parp-1^+/+ ^and Parp-1^-/- ^T cells**. (A) Splenic T cells from Parp-1^+/+ ^(white bars) and Parp-1^-/- ^(black bars) were stimulated with plate-bound anti-CD3 mAb alone or in combination with anti-CD28 mAb for 24 h, 48 h and 72 h. Proliferation was measured by ^3^H-thymidine uptake during the last 12 h of culture. Data are representative of 3 separate experiments carried on in triplicate. Results represent the mean value ± SD. *P < 0.05 in t-test. (B) Apoptosis of T cells from Parp-1^+/+ ^(white bars) and Parp-1^-/- ^(black bars) was detected by FITC-Annexin-V and Propidium iodide staining and flow cytometric analysis at 24 hours after stimulation. Results represent the mean value ± SD of two independent experiments.

T cell proliferation is a hallmark of T cell activation. To determine whether PARP-1-dependent transcriptional regulation influences T cell function, we analyzed the proliferation response of Parp-1^-/- ^and control T cells after in vitro stimulation with anti-CD3 mAb alone or in combination with anti-CD28 mAb. Figure 5A shows a modest, but statistically significant, decrease in T cell proliferation upon stimulation in Parp-1^-/- ^compared to control cells while T cells from both genotypes show similar susceptibility to death upon activation suggesting an intrinsic defect in proliferation in the absence of PARP-1 (Fig. 5B). The role of PARP-1 in apoptosis is variable, depending on the stimuli and the type of cells. Oliver et al. reported that primary bone marrow Parp-1^-/- ^cells are extremely sensitive to apoptosis induced by an alkylating agent but not by a topoisomerase I inhibitor CPT-11 or by interleukin-3 removal [32]. However, Wang et al. reported that Parp-1^-/- ^cells lymphoid cells display normal apoptotic sensitivity after treatment with various apoptotic agents [33]. In addition, recently we have demonstrated that PARP-2 deficiency affects CD4^+^CD8^+ ^apoptosis susceptibility while we did not found any effect on Parp-1^-/- ^mice [34].

Although proliferation is a complex process that requires modulation of different genes, some of the PARP-1 dependent genes identified in this analysis may help to understand the mutant phenotypes observed, thereby establishing a possible link between the transcriptome and the proliferation phenotype. Thus, the reduced expression of Slamf1 and Rel [35] genes in the absence of PARP-1 might be related with the reduced proliferative response of Parp-1^-/- ^T cells.

## Conclusion

This study provides new insights into the role of PARP-1 in T cell activation by modulating gene expression. Using a microarray based approach, we identified 129 genes whose expression in response to anti-CD3 stimulation is PARP-1 dependent. Likewise, expression of 203 genes upon anti-CD3 + anti-CD28 stimulation was also dependent of PARP-1. By using automatic functional annotation of PARP-1-dependent genes we identified GO terms that were significantly over-represented in each activation condition (anti-CD3 alone or anti-CD3 in combination with anti-CD28), including a group of genes involved in lymph immune response. Functional dissection should help to unravel the remaining mysteries of PARP-1 as a signalling molecule at the nuclear level in T cells and might be essential for future development of new therapeutic approaches targeting PARP-1 in the acquired immune response.

## Methods

### Mice

The generation of Parp-1^-/- ^mouse strain (129/Sv × C57BL/6J) has been described previously [36]. Parp-1^-/- ^mice were back-crossed into the C57BL/6J background for seven generations. The animals were kept under standardised conditions and tap water and mouse chow were provided *ad libitum*. All experiments were performed with splenic T cells from 8–10 week old mice on a C57BL/6J background. The experimental procedure was approved by the ethics committee of the University of Murcia and performed in accordance with institutional animal care guidelines that are in compliance with the National Institutes of Health's Guide for the Care and Use of Laboratory Animals.

### Isolation and stimulation of primary T cells

Primary T cells were isolated from spleens of Parp-1^-/- ^and Parp-1^+/+ ^mice by magnetic depletion of non-T cells using a MACS Pan-T Cell isolation kit, according to the manufacturer's instructions (Miltenyi Biotec, Bergisch Gladbach, Germany). Purity was assessed by flow cytometry analysis using antibodies against CD3, CD4 and CD8 and all preparations were more than 98% pure T cells. The cells were activated with plate-bound hamster anti-mouse CD3 (clone 145-2C11) (5 μg/ml) in the absence or the presence of hamster anti-mouse CD28 (clone 37.51) (5 μg/ml) both from BD PharMingen (San Diego, CA) and cultured in RPMI 1640 medium (BioWhittaker) supplemented with 10% FCS, 2 mM L-glutamine, 5 × 10^-5 ^M 2-mercaptoethanol, 2.5 μg/ml fungizone, 100 IU/ml penicillin, and 10 μg/ml streptomycin, all from Sigma (St. Louis, MO).

### Flow cytometry

Cell suspensions were washed in PBS, resuspended in PBS containing 0.5% BSA and incubated with antibodies on ice for 30 min. Incubation with biotin-labelled antibodies was followed by incubation with streptavidin-Tri-color conjugate (Caltag, Burlingame, CA). Phycoerythrin (PE)-conjugated rat anti-mouse CD4 (GK1.5), fluorescein isothiocyanate (FITC)-conjugated rat anti-mouse CD8 (53-6.7) and the biotin-conjugated hamster anti-mouse CD3 (145-2C11) were from BD PharMingen. For apoptosis detection, cells were washed with PBS and the presence of apoptotic cells was analyzed using the annexin V-FITC apoptosis detection kit (Pharmigen) according to the manufacturer's instructions. All cell analysis was performed with a FACSort flow cytometer and CellQuest software (BD).

### Microarray analysis

T cells were pooled from either 4 independent Parp-1^+/+ ^mice or 4 independent Parp-1^-/- ^mice (every pool composed of equal amounts of cells from each mice) in each independent experiment. Cells were stimulated with plate-bound anti-CD3 alone or in combination with anti-CD28 for 3.5 h. Total RNA was isolated from cells by using a Rneasy Total RNA Isolation kit (Qiagen, Valencia, CA) following the manufacturer's instructions. Total RNA (8 μg) was subjected to reverse transcription with Superscript (Life Technologies, Grand Island, NY), using a T7-(dT)_24 _primer containing a T7 RNA polymerase promoter site. Biotinylated complementary RNA was made from 1 μg of cDNA and then fragmented to approximately 50–100 nucleotides, following Affymetrix's instructions. 15 micrograms of the in vitro transcripts with appropriate controls and spikes were hybridized for 16 h at 45°C with constant rotation at 60 rpm to an Affymetrix MOE-430A 2.0 array (Affymetrix, Santa Clara, CA), which contains probes for 22600 known genes and expressed sequences tags (ESTs). Chips were washed and stained by using the EukGE-WS2v4 protocol on an Affymetrix fluidics station. The stain included streptavidin-phycoerythrin (10 μg/ml) (Molecular Probes, Eugene, OR) and biotinylated goat anti-streptavidin (3 μg/ml) (Vector Laboratories, Burlingame, CA). Chips were scanned with Agilent Gene Array Scanner and visualized and analyzed using Affymetrix software (Affymetrix Microarray Suite 5.0; Affymetrix Data Mining Tool 3.0; Affymetrix MicroDB 3.0). Expression values of transcripts were normalized, according to the total intensity on the chip. Only those differences in RNA abundance that were reproducible in at least two independent experiments with different batches of cells (each batch represents a pool of 4 mice) and representing a change of 1.5-fold or greater (p-value <0.05) were considered. The utility of pooling biological samples in microarray experiments for measurement of groups of individuals using few arrays when primary interest is not on the individual, but rather on characteristics of the population from which certain individuals are obtained, has been previously reported [37].

### Quantitative RT-PCR

Total RNA was isolated from the cells by using the Rneasy Total RNA Isolation kit (Qiagen), with on-column DNase I (Qiagen) digestion, following the manufacturer's instructions. cDNA synthesis, and Quantitative real-time PCR was performed as previously described [15]. Two independent experiments with different batches of cells were carried out. Each batch represents a pool of 3 independent mice with equal amount of cells from each mouse. Specific primers for the different genes are described in Table 2. Each gene was normalized to the Gapdh gene. This gene has been shown to remain unchanged under our experimental conditions in our microarray analysis. In addition, it has been shown that at early activation time (< 4 hours), Gapdh expression remains unchanged in response to anti-CD3 in human T cells [38]. The relative changes in gene expression were calculated using the 2-^ΔΔCT ^method [39]. Briefly, cycle threshold (C_T_) value means the number of PCR cycles required for the detection of fluorescence signal to exceed a fixed threshold. The data were analysed using ΔΔC_T _= C_T target _- C_T gapdh_. With this calculation ΔΔC_T _equals to log_2 _ratio, therefore value 1 of ΔΔC_T _corresponds to a 2-fold change.

**Table 2:** Primer sets used for Quantitative real time PCR analysis

Gene	Forward primer	Reverse primer
Egr-2	GCCAAGGCCGTAGACAAAATC	CCACTCCGTTCATCTGGTCA
Nr4a1	TGAGACCCTATCCTCCAGCG	TCTGGCTCGGGGAGAAGTG
Prnp	ATGGCGAACCTTGGCTACTG	CCTGAGGTGGGTAACGGTTG
Il12rb1	ATGGCTGCTGCGTTGAGAA	AGCACTCATAGTCTGTCTTGGA
Il-4	GGTCTCAACCCCCAGCTAGT	GCCGATGATCTCTCTCAAGTGAT
Ccl4	TTCCTGCTGTTTCTCTTACACCT	CTGTCTGCCTCTTTTGGTCAG
Rel	CAGAATTTGGACCAGAACGCA	TGCTGTTCACCCACATTGAAAG
Slamf1	CAGAAATCAGGGCCTCAAGAG	CACTGGCATAAACTGTGGTGG
Gbp7	TCCTGTGTGCCTAGTGGAAAA	CAAGCGGTTCATCAAGTAGGAT
Ccl9	CCCTCTCCTTCCTCATTCTTACA	AGTCTTGAAAGCCCATGTGAAA

### Proliferation assay

T cells were seeded in 96-well plates (2 × 10^5 ^cells/well) and stimulated with anti-CD3 alone or in combination with anti-CD28 mAb as described above. The cultures were conducted for 24 h, 48 h and 72 h and pulsed with 1 μCi [^3^H]thymidine (Moravek Biochemicals, Brea, CA) for the last 12 h of culture. Radioactivity incorporated into DNA was measured by liquid scintillation counting. Experiments were performed in triplicate.

### ELISA

To determine the amount of secreted IL-4, cells were seeded in 96-well plates at subclonfluent density overnight. The enzyme-linked immunosorbent assays (ELISA) for IL-4 was carried out using cell-free supernatants from untreated or anti-CD3 or anti-CD3 + anti-CD28 treated cells, according to the manufacturer's protocol (Quantikine^® ^mouse IL-4 immunoassay, R&D Systems, Minneapolis, MN).

### Statistical analysis

Cel files obtained from MAS software were normalised using robust multiarray analysis (RMA). RMA has been demonstrated to provide improved precision over the default algorithms provided by Affymetrix, which generates MAS5.0 signals [40]. Differential expression was assessed by using linear models and empirical Bayes moderated F-statistics [41]. An interaction term was computed to know which genes respond differently to each treatment (anti-CD3 or anti-CD3-CD28) in PARP-1 mutant phenotypes compared to wild-type cells. Selected genes were subjected to a GO enrichment analysis computing the ratio of observed vs number of genes expected for each GO term, using a Fisher Test from the GO-STATS statistical package. All these analyses were performed using the Bioconductor Package run in the R statistical programming environment [42]. IL-4 production and T cell proliferation results are presented as mean values ± SD. Statistical differences between groups were evaluated by the two-tailed Student's t test. A p-value < 0.05 was considered as statistically significant.

## Additional data files

The following additional data are available with the online version of this paper. Additional data files 1 and 2 are tables listing the genes whose expression at basal level was higher in Parp-1^-/- ^than in Parp-1^+/+^T cells and the genes whose expression was lower in Parp-1^-/- ^compared to Parp-1^+/+ ^T cells, respectively. Additional data files 3 and 4 are tables listing the PARP-1 dependent genes in T cells upon stimulation with anti-CD3 mAb alone or in combination with anti-CD28 mAb, respectively.

## Supplementary Material

Additional file 1 Specific genes expressed in Parp-1^-/- ^vs Parp-1^+/+ ^T cells. Table listing the genes whose expression at basal level was higher in Parp-1^-/- ^than in Parp-1^+/+ ^T cells. Click here for file

Additional file 2 Specific genes expressed in Parp-1^+/+ ^vs Parp-1^-/- ^T cells. Table listing the genes whose expression at basal level was lower in Parp-1^-/- ^compared to Parp-1^+/+ ^T cells. Click here for file

Additional file 3 PARP-1 dependent genes in T cells upon stimulation with anti-CD3. Table listing the PARP-1 dependent genes in T cells upon stimulation with anti-CD3 mAb alone. Click here for file

Additional file 4 PARP-1 dependent genes in T cells upon stimulation with anti-CD3 in the presence of co-stimulation. Table listing the PARP-1 dependent genes in T cells upon stimulation with anti-CD3 mAb in combination with anti-CD28 mAb. Click here for file
